# Pulmonary retention of primed neutrophils: a novel protective host response, which is impaired in the acute respiratory distress syndrome

**DOI:** 10.1136/thoraxjnl-2013-204742

**Published:** 2014-04-04

**Authors:** Charlotte Summers, Nanak R Singh, Jessica F White, Iain M Mackenzie, Andrew Johnston, Chandra Solanki, K K Balan, A Michael Peters, Edwin R Chilvers

**Affiliations:** 1 Department of Medicine, University of Cambridge School of Clinical Medicine, Cambridge, UK; 2 Department of Anaesthesia, Addenbrooke's Hospital, Cambridge University Hospitals NHS Foundation Trust, Cambridge, UK; 3 Department of Nuclear Medicine, Addenbrooke's Hospital, Cambridge University Hospitals NHS Foundation Trust, Cambridge, UK; 4 Department of Radiology, University of Cambridge School of Clinical Medicine, Cambridge, UK

**Keywords:** ARDS, Neutrophil Biology

## Abstract

**Rationale:**

Acute respiratory distress syndrome (ARDS) affects over 200 000 people annually in the USA. Despite causing severe, and often refractory, hypoxaemia, the high mortality and long-term morbidity of ARDS results mainly from extra-pulmonary organ failure; however the mechanism for this organ crosstalk has not been determined.

**Methods:**

Using autologous radiolabelled neutrophils we investigated the pulmonary transit of primed and unprimed neutrophils in humans. Flow cytometry of whole blood samples was used to assess transpulmonary neutrophil priming gradients in patients with ARDS, sepsis and perioperative controls.

**Main results:**

Unprimed neutrophils passed through the lungs with a transit time of 14.2 s, only 2.3 s slower than erythrocytes, and with <5% first-pass retention. Over 97% of neutrophils primed ex vivo with granulocyte macrophage colony-stimulating factor were retained on first pass, with 48% still remaining in the lungs at 40 min. Neutrophils exposed to platelet-activating factor were initially retained but subsequently released such that only 14% remained in the lungs at 40 min. Significant transpulmonary gradients of neutrophil CD62L cell surface expression were observed in ARDS compared with perioperative controls and patients with sepsis.

**Conclusions:**

We demonstrated minimal delay and retention of unprimed neutrophils transiting the healthy human pulmonary vasculature, but marked retention of primed neutrophils; these latter cells then ‘deprime’ and are re-released into the systemic circulation. Further, we show that this physiological depriming mechanism may fail in patients with ARDS, resulting in increased numbers of primed neutrophils within the systemic circulation. This identifies a potential mechanism for the remote organ damage observed in patients with ARDS.

**Key messages**

**What is the key question?**

- How does acute respiratory distress syndrome (ARDS) result in extra-pulmonary organ failure?

**What is the bottom line?**

- The healthy human lung may play a critical role in host defence by selectively retaining primed neutrophils, facilitating their depriming and re-releasing them into the systemic circulation in a quiescent state; this depriming mechanism appears to fail in patients with ARDS, resulting in exposure of remote organs to primed neutrophils.

**Why read on?**

- We demonstrate for the first time that the healthy human lung can retain primed neutrophils, facilitate their depriming and later re-release them into the systemic circulation, and that in patients with ARDS this depriming mechanism appears to fail, resulting in elevated levels of primed neutrophils within the systemic circulation. This provides a potent mechanism for the extra-pulmonary organ damage occurring in ARDS.

## Introduction

Acute respiratory distress syndrome (ARDS) affects 200 000 people each year in the USA, and has a mortality rate of approximately 40%.^1^ Due to alterations in demographic factors, it has been estimated that the incidence of ARDS will climb to 335 000 cases per annum by 2030.^2^ Despite causing severe hypoxaemic respiratory failure, most patients with ARDS die as a consequence of non-pulmonary organ failure.^1^ Recently it has been established that even survivors of ARDS have significant long-term extra-pulmonary organ dysfunction.^3^ ^4^ The clinical observation that patients with hypoxemic respiratory failure acquire significant remote organ dysfunction has led to interest in the concept of organ ‘crosstalk’.

Several experimental and clinical studies provide evidence to support the concept that lung damage may propagate to remote organs. However, the mechanisms by which this happens are not yet established. Imai and colleagues^5^ demonstrated that injurious mechanical ventilation may lead to epithelial cell apoptosis in remote organs such as the kidney, which they propose is induced by factors released by the lung. Similarly, Guery *et al*^6^ reported elevated plasma tumour necrosis factor α levels and gut permeability in a ventilator-induced lung injury model, supporting the hypothesis of crosstalk between the lungs and the gastrointestinal tract. There is also similar evidence for lung–brain interaction.^7^ While humeral factors have been suggested to mediate such interactions, cellular mechanisms may also operate.

Neutrophils are the most abundant circulating white cells in man, and are key effectors of the innate immune response. In contrast, inappropriate accumulation, or activation, of these cells, and/or their delayed clearance, has been linked to several disease states, including ARDS.^8^ The extreme histotoxic potential of neutrophils dictates the need for safety mechanisms to prevent their inadvertent activation. One such mechanism is priming. Neutrophil priming refers to the process whereby exposure of these cells to a variety of inflammatory mediators or physico-chemical perturbations increases subsequent agonist-induced responses. Priming has direct effects on respiratory burst generation, neutrophil shape, deformability, integrin expressio, and longevity, and as a consequence has a profound impact on the rheological, adhesive and survival properties of these cells.^9^ Most importantly, priming has been shown to be a prerequisite for neutrophil-mediated tissue injury; indeed the recruitment of large numbers of primed ‘hyper-responsive’ neutrophils to the lung is thought to play a critical role in the genesis of ARDS.^10^ ^11^

We provide evidence that the healthy pulmonary vasculature may play an important role in host defence by selectively retaining circulating primed neutrophils, facilitating their ‘depriming’, and later releasing them back into the systemic circulation in a quiescent state. We also demonstrate that this depriming mechanism appears to fail in patients with ARDS, leading to elevated levels of primed neutrophils in the systemic circulation, thus providing a potent mechanism for remote organ damage.

## Materials and methods

Two independent methods were used to examine the transit of radiolabelled autologous neutrophils across the lungs of human subjects. All subjects had normal spirometry, no pulmonary symptoms and were non-smokers. Informed consent was obtained in all cases, and the Cambridge and Hertfordshire Research Ethics Committees approved the study protocols (08/H0306/17; 03/385; 08/H0311/62).

### Neutrophil isolation and radiolabelling

Neutrophils were purified from peripheral venous blood using lipopolysaccharide-free discontinuous plasma-Percoll gradients and radiolabelled with either ^111^indium tropolonate or ^99m^technetium-HMPAO, in the presence of autologous plasma, as described previously.^12^ ^13^ Autologous erythrocytes were purified and labelled with ^99m^technetium (as pertechnetate). All cells were resuspended in 100% autologous plasma for reinjection.

### Ex vivo priming of neutrophils

Neutrophils, isolated and radiolabelled as above, were resuspended in autologous plasma and stimulated at 37°C with granulocyte macrophage colony-stimulating factor (GM-CSF) (100 ng/mL) or platelet-activating factor (PAF) (1 μM) for 5 min (PAF-primed neutrophils) or 30 min (deprimed neutrophils). The cells were then washed with autologous plasma (150 g, 5 min at room temperature) and resuspended in plasma.

### Measurement of neutrophil transit time using γ scintigraphy

**Figure 1:**
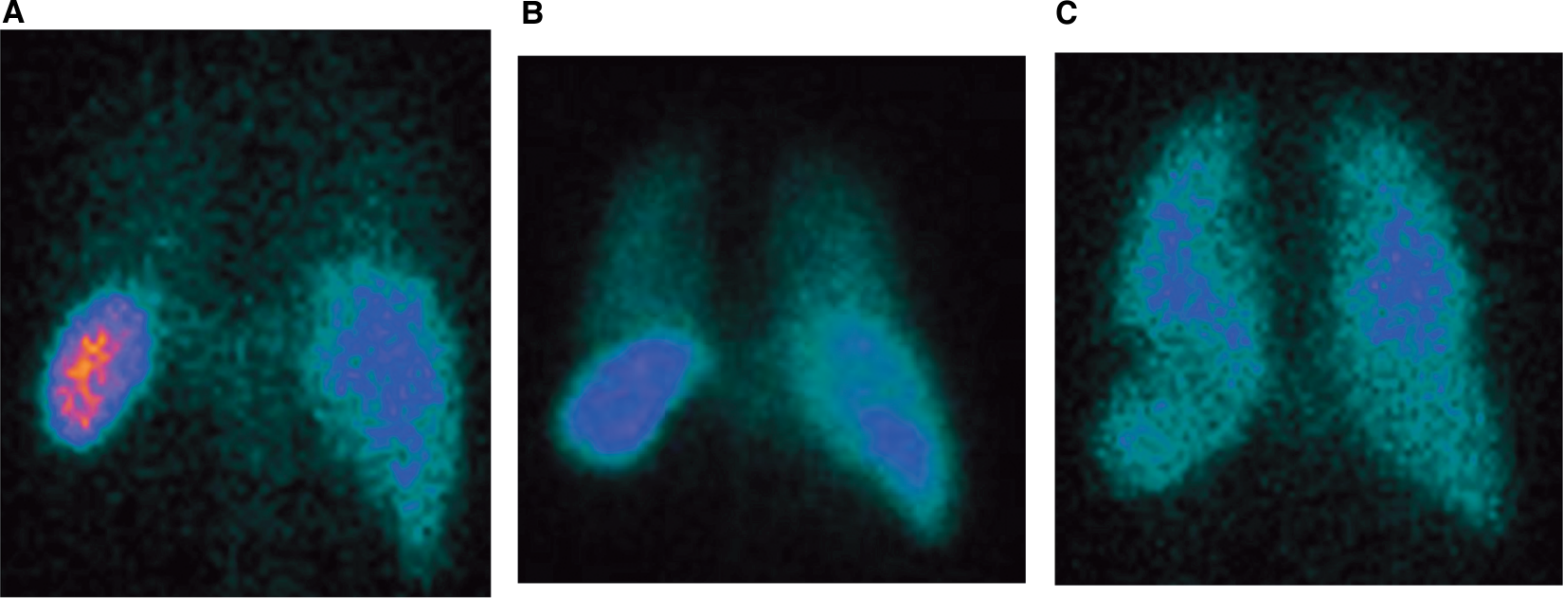
Representative images obtained from the posterior head of γ camera 40 min after injection of autologous human neutrophils. Autologous ^99m^technetium (Tc)-labelled neutrophils were primed with granulocyte macrophage colony-stimulating factor (GM-CSF) (100 ng/mL, 30 min, n=8), platelet-activating factor (PAF) (1 μM, 5 min, n=5; or 30 min, n=6), or control (phosphate-buffered saline (PBS), 5 min, n=8), washed twice with autologous plasma (150 g, 5 min) and resuspended in plasma, before being reinjected into the left antecubital fossa of healthy spontaneously breathing volunteers, lying supine in a dual-headed γ camera. Images were acquired at 1/s for 2 min, followed by 1/20 s for 38 min, from the time of injection. (A–C) Shows representative images from the posterior head of the γ camera for unprimed (A), deprimed (PAF for 30 min; B) and GM-CSF primed (C) autologous neutrophils 40 min post injection into the left antecubital fossa. ^111^In, ^111^indium tropolonate; RBC, red blood cell; ROI, region of interest.

^99m^Technetium-labelled neutrophils were injected into a left antecubital fossa vein of spontaneously breathing adults in the supine position. Imaging was performed using an Elscint double-headed γ camera fitted with medium energy collimators. To assess the pulmonary transit of neutrophils, a dynamic sequence was acquired at a frame rate of 1/s for 2 min, followed by 1 frame/20 s for a further 38 min. Representative images from the posterior head of the γ camera, acquired 40 min after injection, are shown in figure 1. Regions of interest (ROIs) were drawn around the right ventricle and lungs, and the mean counts per pixel recorded. A γ variate was fitted using a least squares residual method to simulate the first pass time–concentration curve for neutrophils across the lung. This experiment was undertaken using unprimed neutrophils (n=8), GM-CSF primed neutrophils (n=8), PAF primed neutrophils (n=5) and PAF deprimed neutrophils (n=6). The demographics of the subjects participating are shown in table 1.

**Table 1:** Demographics of subjects undergoing gamma scintigraphy

	All	Control	GM-CSF	PAF primed	PAF deprimed
N	27	8	8	5	6
Number of men	9	2	4	1	2
Age in years	50.5 (46.8–59.3)	50.0 (47.0–62.8)	52.5 (45.2–61.0)	50.0 (49.0–55.5)	55.0 (45.5–56.5)
Percentage predicted FEV_1_/FVC	78.1 (72.5–82.3)	78.2 (72.9–80.0)	76.9 (73.0–81.9)	80.4 (73.0–83.4)	78.0 (73.7–83.7)

Data shown as median (IQR).

GM-CSF, granulocyte macrophage colony-stimulating factor; PAF, platelet-activating factor.

### Measurement of neutrophil transit time using outflow tract sampling

^111^Indium-tropolonate-labelled neutrophils and ^99m^technetium-labelled erythrocytes were mixed and reinjected as a single bolus into the right internal jugular vein of six anaesthetised adults (table 2), prior to remote surgery. Using a high fidelity peristaltic pump (LiDCO, UK) and fraction counter (Pharmacia LKB FRAC-100), blood samples were taken every 3.6 s for 4 min from a left radial arterial catheter. Blood ^111^indium and ^99m^iechnetium concentrations were measured in the collected fractions using a Wallac Wizard 3–1480 Automatic Gamma Counter. Samples were recounted after 10 ^99m^technetium half lives had elapsed (t_1/2_=6.02 h) to ensure accuracy of the indium counting (t_1/2_^111^In=67.4 h). Measured activity values were corrected for background radiation, radioisotope decay and crosstalk, before being expressed as a fraction of the injected activity. To remove the effects of cell recirculation and to simulate a first-pass transit curve, the time–concentration curves were fitted with γ variate functions using a least squares residual method. Mean transit times for neutrophils and erythrocytes were derived from the areas under the first-pass transit curves.

**Table 2:** Demographics of subjects undergoing outflow tract sampling

Surgical procedure	Age (years)	Sex	Cardiac output (L/min)	Peripheral blood neutrophil count (10^9^/L)
Radical cystectomy	69	Male	5.5	2.9
Radical cystectomy	80	Male	3.1	5.0
Radical cystectomy	76	Male	5.9	7.6
Radical cystectomy	65	Female	5.7	4.4
Oesophagectomy	53	Male	5.7	5.5
Oesophagectomy	75	Male	5.2	2.6

All pulmonary transit measurements were undertaken with subjects supine, receiving 6 ml/kg predicted body weight tidal volume ventilation, positive end-expiratory pressure of 5 cm H_2_O and FiO_2_ 0.5.

The pulmonary retention fraction of neutrophils was calculated using the methodology of Hogg *et al.*^14^
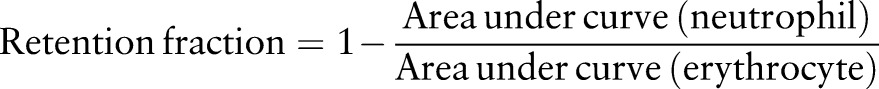


### Assessment of neutrophil priming gradients

Paired samples of whole blood were simultaneously obtained from the radial artery and internal jugular veins of critically ill patients with systemic sepsis with no evidence of pulmonary involvement (n=6), ARDS (diagnosed according to The ARDS Definition Task Force^15^; n=8), and perioperative control patients (n=5) (table 3). Absolute neutrophil count was measured using a Coulter DXH automated counter, whilst neutrophil shape change, CD11b and CD62L cell surface expression were analysed using a no-lysis whole blood flow cytometry method, based on a previous publication.^16^ Gradients were expressed as a ratio of the arterial value over the venous value. The raw data are provided in online supplementary table S1.

**Table 3:** Demographic data of transpulmonary gradient study patients

Group	Age (years)	Sex	P/F ratio (kPa)	Diagnosis
Perioperative controls	62	Male	78.2	Oesophagectomy for malignancy
	29	Male	81.0	Oesophagectomy for non-malignant constriction
	70	Female	74.8	Oesophagectomy malignancy
	70	Male	82.7	Oesophagectomy for malignancy
	48	Male	88.1	Oesophagectomy malignancy
Sepsis	48	Female	40.0	Chronic liver disease
	84	Male	44.4	Gram-negative bacteraemia
	70	Female	44	Streptococcal bacteraemia
	66	Female	48.2	Biliary sepsis
	55	Female	42.8	Large bowel perforation
	79	Female	53.7	Small bowel perforation
ARDS	29	Female	13.3	H1N1 influenza
	39	Male	23.3	Polytrauma
	79	Male	14.9	Septic shock of unknown source
	75	Male	13.0	Community acquired pneumonia
	48	Female	14.0	Chronic liver disease
	84	Male	13.7	Necrotising fasciitis
	51	Male	15.7	Hospital-acquired pneumonia
	71	Male	17	Biliary sepsis

ARDS, acute respiratory distress syndrome.

### Statistical analysis

Because of small sample sizes, and because the normality of distribution of the data could neither be assumed nor tested, non-parametric methods were used for statistical analysis. A p value of <0.05 was considered significant. Data are expressed as median (IQR) unless otherwise specified.

## Results

### Less than 5% of unprimed neutrophils are retained by the healthy pulmonary vasculature

**Figure 2:**
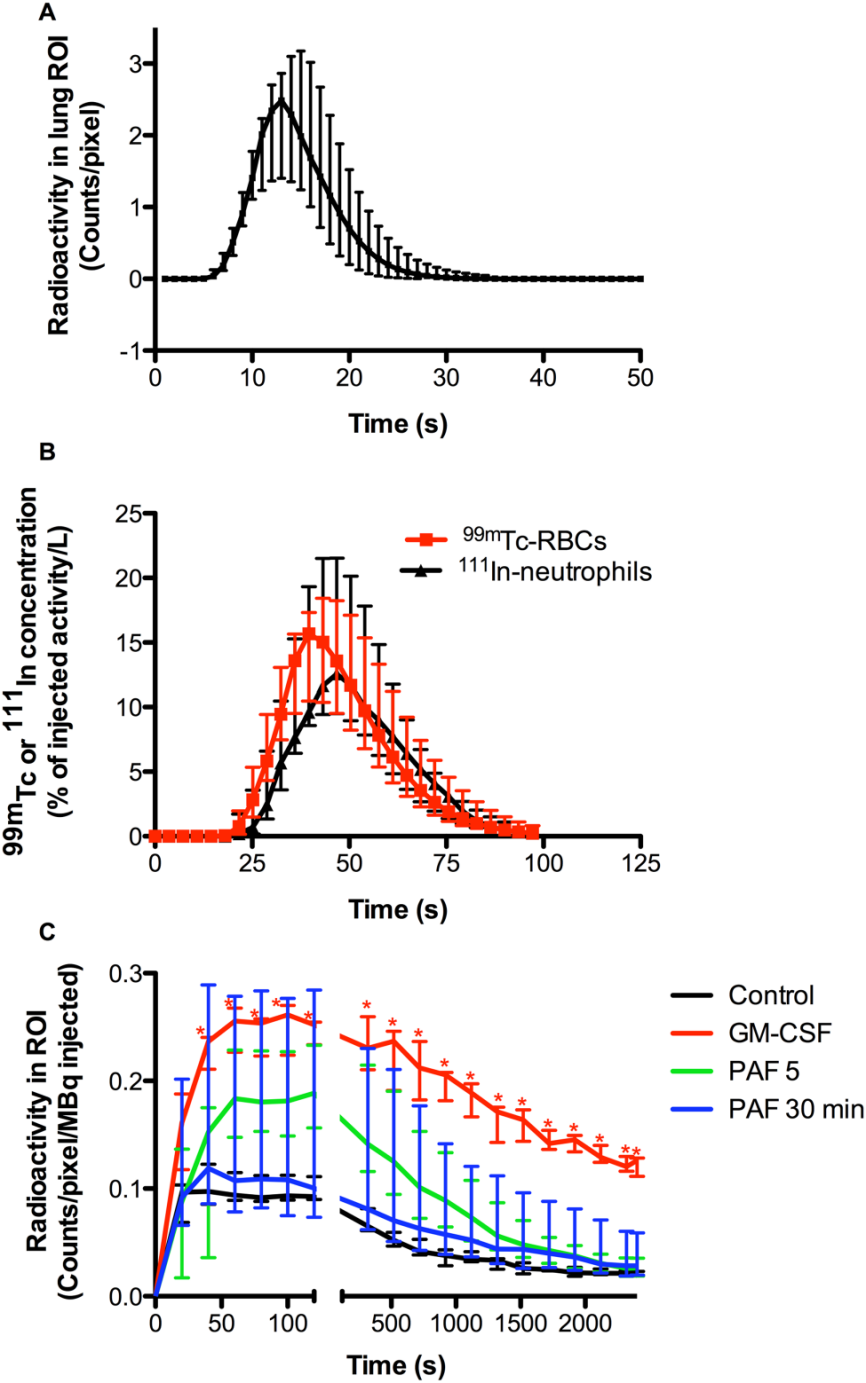
Effect of neutrophil priming on pulmonary transit kinetics. Autologous ^99m^technetium-labelled neutrophils were primed with granulocyte macrophage colony-stimulating factor (100 ng/mL, 30 min, n=8), platelet-activating factor (1 μM, 5 min, n=5; or 30 min, n=6) or control (phosphate-buffered saline, 5 min, n=8), washed twice with autologous plasma (150 g, 5 min) and resuspended in plasma, before being reinjected into the left antecubital fossa of healthy spontaneously breathing volunteers, lying supine in a dual-headed γ camera. Images were acquired at 1/s for 2 min, followed by 1/20 s for 38 min, from the time of injection. Regions of interest were drawn around the lungs and the average count per pixel recorded. The data were corrected for radioisotope decay and plotted against time. A γ variate was fitted to the control data to simulate a first-pass transit curve for unprimed neutrophils (A), from which a mean transit time of 14.18 s (14.06–14.61 s) was derived. (C) Shows the median and IQR of data obtained from all 27 independent experiments. *Represents p<0.05 compared with control. To validate our findings regarding the transit kinetics of unprimed neutrophils, autologous ^99m^technetium-labelled erythrocytes and ^111^indium-labelled neutrophils were mixed with lithium chloride and injected as a single bolus into the right internal jugular veins of patients with healthy lungs (normal spirometry and thoracic CT) under surgical anaesthesia. Starting immediately prior to injection, continuous blood sampling was undertaken from the left radial artery (collected in 3.6 s fractions) using a peristaltic pump and fraction collector. Blood ^99m^technetium and ^111^indium activity was measured, with appropriate corrections for background, crosstalk and radionuclide decay, and expressed as a fraction of the administered activities. Each first-pass curve was fitted with a γ variate function to calculate the area under the first-pass curve and the difference in lung mean transit times of erythrocytes and neutrophils. (B) Shows the median and IQR of six independent patient studies.

Gamma scintigraphy of ^111^indium-labelled neutrophils, prepared in the continuous presence of autologous plasma, reinjected into healthy volunteers demonstrated that the transit time of unprimed neutrophils across the pulmonary circulation was 14.2 s (14.1–14.6 s) (n=8; figure 2A). All lung washout curves were mono-exponential, and following first pass there was no detectable accumulation of neutrophils within the lungs over the subsequent 40 min (data not shown).

Given that the transit time of unprimed neutrophils in our experiments was significantly faster than values reported in the literature, we undertook additional validation of our findings, using a second approach involving a novel outflow-tract sampling method. In these experiments the transit time of unprimed neutrophils from the right internal jugular vein to the left radial artery was found to be only 2.3 s (0.9–4.2 s) longer than that of admixed ^99m^technetium-labelled erythrocytes, and less than 5% of the injected neutrophils were retained in the lungs on first pass (4.7% (0.5–9.2%); n=6; figure 2B). The washout curve of neutrophils from the lung was again mono-exponential, suggesting that the vast majority of neutrophils traversed the pulmonary vasculature with only minimal delay. Additional data, obtained in a further four subjects who were given radiolabelled ‘mixed leukocytes’ that had not been exposed to the Percoll-plasma isolation procedure, revealed a mixed leukocyte delay, compared with simultaneous erythrocyte transit, of 3.1 s (2.1–4.9 s), supporting the view that there were no detrimental effects of the neutrophil isolation technique used.

### Neutrophil priming causes reversible retention of neutrophils by the pulmonary vasculature

Remarkably, 96.5% (96.0–98.7%) of autologous radiolabelled neutrophils primed ex vivo with GM-CSF (n=8) were retained on first pass across the lung, with no evidence of washout over the first 2 min. Subsequently, these neutrophils were released slowly from the pulmonary vascular bed, such that 48.3% (42.5–59.7%) of the maximal ^99m^technetium signal detected within the lung ROI was still present 40 min post injection. A total of 12.9% (11.2–17.4%) of the injected GM-CSF primed neutrophils were recovered from the peripheral blood 40 min post injection.

We have shown previously in vitro that, in contrast to GM-CSF, PAF can induce neutrophil priming that is fully reversible, with maximal priming effect at 5 min and a recovery to an unprimed state by 30 min.^17^ Neutrophils exposed to PAF ex vivo for 5 min and reinjected into healthy volunteers were initially retained in the lungs, as observed with GM-CSF primed cells, but were subsequently released far more rapidly, such that only 13.9% (13.3–14.3%) of the maximal signal was still observed at 40 min (n=5). This would fit predictions based on previously published in vitro observations regarding the different time courses of priming responses elicited by different agonists.^18^ Neutrophils exposed to PAF for 30 min (by which time we would predict they would be fully ‘deprimed’) appeared initially to be released from the lung more rapidly than acutely PAF primed cells, although this was not statistically significant, raising the possibility that for a single priming agent, pulmonary neutrophil retention may scale with the priming intensity (n=6). The dataset is summarised in figure 2C. The 40 min peripheral blood recovery of neutrophils exposed to PAF for 30 min was 27.6% (26.4–31.4%) compared with values for unprimed cells of 37.3% (33.1–39.9%).

### ARDS is associated with a failure of neutrophil depriming and increased systemic levels of neutrophil priming

Our data demonstrating the ability of the pulmonary vasculature to retain primed neutrophils and release them back into the circulation at a later point led us to the hypothesis that the pulmonary vasculature may play an important role in host defence, protecting the systemic circulation from the histotoxic effects of primed neutrophils by trapping and depriming neutrophils, and further, that should this mechanism fail, neutrophilic pulmonary inflammation, such as that seen in ARDS, may result.

To test this hypothesis, simultaneous paired blood samples were taken from the internal jugular vein and radial artery of patients with sepsis, ARDS and perioperative controls. We used assays specifically designed to evaluate neutrophil number and priming status in whole blood to avoid further ex vivo changes. Primed neutrophils were considered to be shape changed (as assessed by mean forward scatter), CD11b^high^/CD62L^low^, whilst unprimed neutrophils were non-shape changed and CD11b^low^/CD62L^high^.

**Figure 3:**
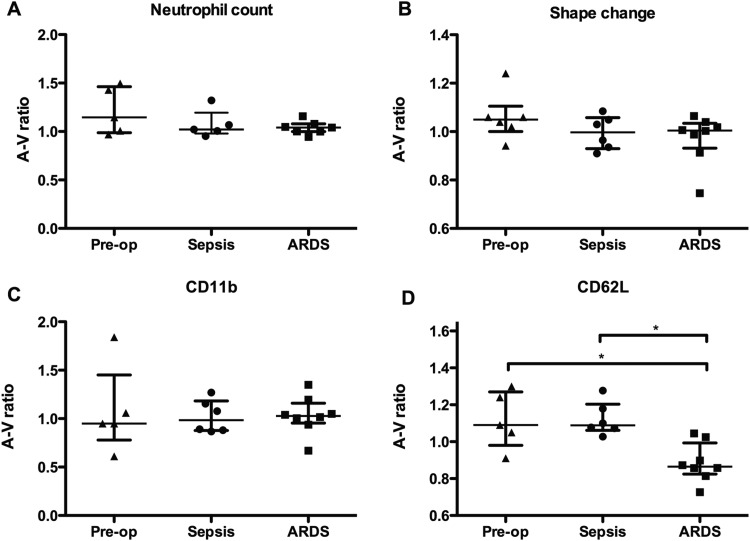
Measurement of neutrophil priming gradients across the lungs. Paired samples of whole blood were obtained from the radial artery and internal jugular veins of critically ill patients with systemic sepsis (n=6), acute respiratory distress syndrome (ARDS) (n=8), or perioperative controls (n=5). Absolute neutrophil count was measured using a Coulter DXH. Neutrophil shape change and cell surface expressions of CD11b and CD62L were measured using no lysis whole blood flow cytometry. Gradients were expressed as the ratio of the arterial value over the venous value. (A) Shows a scatter plot (with mean and IQR) for neutrophil count, (B) shape change (expressed as mean forward scatter), (C) shows CD11b cell surface expression, and (D) shows CD62L cell surface expression. Analysed using Kruskal–Wallis test with Dunn's post hoc comparisons. *Represents p<0.05.

**Figure 4:**
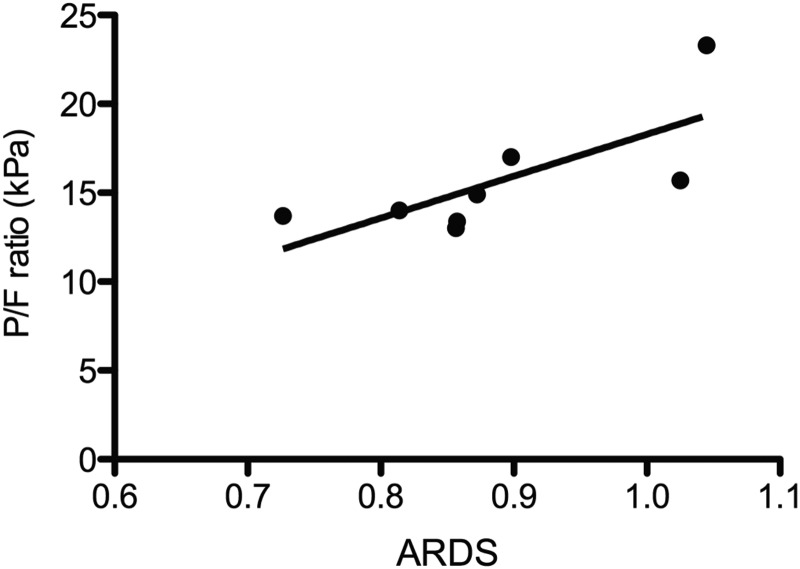
Correlation between A and V gradient of neutrophil CD62L cell surface expression and oxygenation status in patients with acute respiratory distress syndrome (ARDS). Paired samples of whole blood were obtained from the radial artery and internal jugular veins of patients with ARDS (n=8 patients). Neutrophil cell surface expression of CD62L was measured using no lysis whole blood flow cytometry. Gradients were expressed as the ratio of the arterial value over the venous value. The P/F ratio (pO_2_ in arterial blood/fraction of inspired oxygen) was recorded at the time each sample was taken. Figure 4 shows the Spearman correlation between A and V gradient of neutrophil CD62L cell surface expression and P/F ratio (R=0.7857, p<0.05).

We detected no significant gradient across the lungs with respect to absolute neutrophil count, neutrophil shape change, or neutrophil CD11b expression in any of our subject groups (figure 3A–C). However, a significant difference in the transpulmonary gradient of CD62L expression was observed (figure 3D; p<0.05). There was no difference between the transpulmonary gradient of CD62L expression of patients with sepsis and control subjects, suggesting that in these subjects the capacity to deprime neutrophils was intact. In contrast, subjects with ARDS had a significant decrease in transpulmonary gradient of CD62L expression compared with controls (p<0.05), indicating that the lung's capacity to retain and deprime neutrophils may have been compromised. Of interest, the transpulmonary gradient of CD62L expression correlated with the oxygenation status of patients with ARDS (R=0.7857, p<0.05; figure 4).

Online supplementary figure S1 shows the changes in the transpulmonary gradient of CD62L occurring in a patient with sepsis who subsequently developed ARDS.

## Discussion

ARDS affects 200 000 people per annum in the USA and this figure is set to increase over the next 25 years.^2^ A substantial proportion of these patients die as a result of multiorgan failure. The mechanism linking ARDS, a disease characterised by pulmonary inflammation and severe hypoxeamic respiratory failure, to extra-pulmonary organ dysfunction is uncertain, however, clinical and experimental studies support the concept of organ crosstalk.

Neutrophils are key cells in the pathogenesis of ARDS. Clinical studies have shown that neutrophil accumulation within the pulmonary vasculature occurs early in the evolution of ARDS,^19^ and neutrophilic alveolitis is a histological hallmark.^20^ Neutrophilia is common in the bronchoalveolar lavage fluid of patients with ARDS^21^ and the extent of this correlates with clinical outcome.^22^ Animal models also support the importance of neutrophils in ARDS: neutrophil depletion ameliorates the extent of ARDS,^23–27^ and delayed neutrophil apoptosis, or failure of apoptotic cell clearance, is associated with worsened inflammation and increased mortality.^28^ However, the presence of neutrophils per se is not necessarily damaging, with the priming/activation status of these cells being the major determinant of their subsequent injury limiting/inducing behaviour.^29^ ^30^

Using ex vivo primed autologous human neutrophils and γ scintigraphy, we have shown that neutrophil priming can markedly prolong pulmonary transit time in a manner that is priming agent specific. We have also shown that the vast majority of neutrophils, isolated and labelled under conditions that cause minimal activation, pass through the pulmonary circulation with minimal delay. The accumulation of deliberately primed neutrophils within the pulmonary vasculature was reversible and did not result in cell migration into the pulmonary interstitium. Our data suggest that the healthy human pulmonary vasculature may play a role in host defence, protecting the systemic circulation from the toxic effects of primed neutrophils by retaining primed neutrophils and later re-releasing them back into the systemic circulation in a deprimed state. Further, we observed that ARDS was associated with a significantly decreased transpulmonary gradient of CD62L expression, suggesting that a failure of neutrophil depriming may occur in patients with ARDS, resulting in the presence of elevated levels of primed neutrophils within the systemic circulation. The presence of elevated levels of primed neutrophils within the systemic circulation provides a plausible crosstalk mechanism for the extra-pulmonary organ damage observed in patients with ARDS. Additionally, the increased concentrations of circulating cytokines often found in patients with ARDS may cooperate with the circulating primed neutrophils to inflict extra-pulmonary organ damage. An alternative explanation for the finding of the elevated level of circulating primed neutrophils observed in subjects with ARDS is that the lung itself had become a source of neutrophil priming. The transpulmonary gradient of CD62L expression was correlated with the oxygenation status of patients with ARDS.

The ‘catch and release’ of primed neutrophils by the pulmonary vasculature is entirely consistent with previous studies in which intravenously injected lipopolysaccharide and/or N-formyl-neoleucyl-leucyl-phenylalanine (FNLP) caused the accumulation of ^111^indium-labelled neutrophils in the lungs of rabbits.^31^ ^32^ In these experiments the accumulation of primed neutrophils in the lung was also reversible. Our current data are also consistent with a previous study in humans in which PAF inhalation was shown to induce a temporary blood neutropenia associated with increased lung accumulation of ^111^indium-labelled neutrophils.^33^ While intravenous lipopolysaccharide and FNLP models the effects of systemic sepsis and the inhaled PAF study explores more the effect of pulmonary inflammation on previously native neutrophils, the findings of temporary neutrophil sequestration within the pulmonary vasculature is consistent and supports our current work.

Our findings have several limitations. This work used neutrophils primed ex vivo and infused into healthy subjects. It is possible that the data obtained may not reflect the true in vivo situation in an individual with acute inflammation, as theoretically there may be substantial differences between ex vivo and in vivo primed neutrophils; however, we know of no data to support this suggestion. We were not able to quantify the mean pulmonary transit times of primed neutrophils on first pass because their marked retention prevented any attempt to mathematically model the first-pass transit, which relies on some degree of loss of signal following the initial peak. However, as there was very minimal decrease in the lung signal over the first 2 min following injection of either PAF or GM-CSF primed cells, the absolute transit time for these cells is likely to be considerably longer than 2 min compared with our observed transit time of 14.18 s for unprimed neutrophils. The subjects studied in the coinjection experiments were mainly undergoing surgery for abdominal malignancy; whilst none had smoked in the preceding 6 months, and all had normal chest radiographs and lung function, they cannot be considered entirely healthy subjects, unlike the volunteers recruited for the γ scintigraphy studies.

The current data suggesting a very rapid transit of unprimed neutrophils across the lung does not preclude the possibility of a small subpopulation (<5%) of more slowly transiting cells that are not evident beneath the signal generated by the predominantly fast transiting cells. Measuring the transit time of such a population of neutrophils during in vivo human studies is extremely challenging, as it necessitates prevention of any recirculation. All that can be concluded based on the observed retention fraction and the mono-exponential nature of the pulmonary washout curves is that >95% of neutrophils are not delayed within the lung on first pass. In contrast to our experiments, many earlier studies examining neutrophil transit across the lung demonstrating higher pulmonary retention values were conducted in animals which have been shown to have neutrophils that are less deformable than human neutrophils, and a larger fraction of pulmonary capillaries with a diameter less than that of neutrophils.^34^ Further, recovery values based on tritium-labelled cells have also been shown to vary between species.^35^ The majority of earlier studies undertook radiolabelling with indium oxide, which requires the separation of the neutrophils from autologous plasma, thus altering their subsequent in vivo transit kinetics.^12^

In conclusion, we provide evidence that the healthy human pulmonary vasculature may play a role in host defence by retaining primed neutrophils and later releasing them back into the systemic circulation in a deprimed state. We have also shown that ARDS is associated with elevated levels of primed neutrophils within the systemic circulation, suggesting that ARDS may be associated with a failure of neutrophil depriming, thus providing a potential ‘crosstalk’ mechanism for the remote organ damage that is the predominant cause of mortality and long-term morbidity in patients with ARDS.

## Supplementary Material

Web supplement

## References

[1] Matthay MA Zemans RL The acute respiratory distress syndrome: pathogenesis and treatment. Annu Rev Pathol 2011;6:147–63 20936936 10.1146/annurev-pathol-011110-130158 PMC3108259

[2] Rubenfeld GD Cauldwell E Peabody E Incidence and outcomes of acute lung injury. N Eng J Med 2005;353:1685–93 10.1056/NEJMoa050333 16236739

[3] Herridge MS Tansey CM Matte A Functional disability 5 years after acute respiratory distress syndrome. N Engl J Med 2011;364:1293–304 21470008 10.1056/NEJMoa1011802

[4] Hopkins RO Weaver LK Collingridge D Two-tear cognitive, emotional, and quality-of-life outcomes in acute respiratory distress syndrome. Am J Respir Crit Care Med 2005;171:340–7 15542793 10.1164/rccm.200406-763OC

[5] Imai Y Parodo J Kajikawa O Injurious mechanical ventilation and end-organ epithelial cell apoptosis and organ dysfunction in an experimental model of acute respiratory distress syndrome. JAMA 2003;289:2104–12 12709468 10.1001/jama.289.16.2104

[6] Guery BP Welsh DA Viget NB Ventilation-induced lung injury is associated with an increase in gut permeability. Shock 2003;19:559–63 12785012 10.1097/01.shk.0000070738.34700.bf

[7] Quilez ME Lopez-Aguilar J Blanch L Organ crosstalk during acute lung injury, acute respiratory distress syndrome, and mechanical ventilation. Curr Opin Crit Care 2012;18:23–8 22186216 10.1097/MCC.0b013e32834ef3ea

[8] Cowburn AS Condliffe AM Farahi N Advances in neutrophil biology: clinical implications. Chest 2008;134:606–12 18779195 10.1378/chest.08-0422 PMC2827863

[9] Condliffe AM Kitchen E Chilvers ER Neutrophil priming: pathophysiological consequences and underlying mechanisms. Clin Sci (Lond) 1998;94:461–71 9682667 10.1042/cs0940461

[10] Guthrie LA McPhail LC Henson PM Priming of neutrophils for enhanced release of oxygen metabolites by bacterial lipopolysaccharide: evidence for increased activity of the superoxide-producing enzyme. J Exp Med 1984;160:2656–71 10.1084/jem.160.6.1656 PMC2187529 6096475

[11] Zimmerman GA Renzetti AD Hill HR Functional and metabolic activity of granulocytes from patients with adult respiratory distress syndrome: evidence for activated neutrophils in the pulmonary circulation. Am Rev Respir Dis 1983;127:290–300 6830052 10.1164/arrd.1983.127.3.290

[12] Saverymuttu SH Peters AM Danpure H Lung transit of ^111^Indium-labelled granulocytes. Relationship to labelling techniques. Scand J Haematol 1983;30:151–60 6836229 10.1111/j.1600-0609.1983.tb01463.x

[13] Peters AM Danpure H Osman S Clinical experience with 99mTc-hexamethylpropylene-amineoxime for labelling leukocytes and imaging inflammation. Lancet 1986;2:946–9 2877132 10.1016/s0140-6736(86)90601-x

[14] Hogg JC Doerschuk CM Wiggs B Neutrophil retention during a single transit through the pulmonary circulation. J Appl Physiol 1992;73:1683–5 1447121 10.1152/jappl.1992.73.4.1683

[15] The ARDS Definition Task Force. Acute respiratory distress syndrome: the Berlin definition. JAMA 2012;307:2526–33 22797452 10.1001/jama.2012.5669

[16] Alvarez-Larran A Toll T Rives S Assessment of neutrophil activation in whole blood by flow cytometry. Clin Lab Haematol 2005;27:41–6 15686506 10.1111/j.1365-2257.2004.00661.x

[17] Kitchen E Rossi AG Condliffe AM Demonstration of reversible priming of human neutrophils using platelet-activating factor. Blood 1996;88:4330–7 8943870

[18] Condliffe AM Chilvers ER Haslett C Priming differentially regulates neutrophil adhesion molecule expression/function. Immunology 1996;89:105–11 8911147 10.1046/j.1365-2567.1996.d01-711.x PMC1456672

[19] Bachofen M Weibel ER Alterations of the gas exchange apparatus in adult respiratory insufficiency associated with septicemia. Am Rev Respir Dis 1977;116:589–615 921049 10.1164/arrd.1977.116.4.589

[20] Lee WL Downey GP Neutrophil activation and acute lung injury. Curr Opin Crit Care 2001;7:1–7 11373504 10.1097/00075198-200102000-00001

[21] Parsons PE Fowler AA Hyers TM Chemotactic activity in bronchoalveolar lavage fluid from patients with adult respiratory distress syndrome. Am Rev Respir Dis 1985;132:490–3 4037522 10.1164/arrd.1985.132.3.490

[22] Steinberg KP Milberg JA Martin TR Evolution of bronchoalveolar cell populations in the adult respiratory distress syndrome. Am J Respir Crit Care Med 1994;150:113–22 8025736 10.1164/ajrccm.150.1.8025736

[23] Abraham E Carmody A Shenkar R Neutrophils as early immunologic effectors in hemorrhage- or endotoxemia induced acute lung injury. Am J Physiol Lung Cell Mol Physiol 2000;279:L1137–45 11076804 10.1152/ajplung.2000.279.6.L1137

[24] Folkesson HG Matthay MA Hébert CA Acid aspiration-induced lung injury in rabbits is mediated by interleukin-8-dependent mechanisms. J Clin Invest 1995;96:107–16 7615779 10.1172/JCI118009 PMC185178

[25] Shimizu M Hasegawa N Nishimura T Effects of TNF-alpha-converting enzyme inhibition on acute lung injury induced by endotoxin in the rat. Shock 2009;32:535–40 19295482 10.1097/SHK.0b013e3181a2adb7

[26] Kawabata K Hagio T Matsumoto S Delayed neutrophil elastase inhibition prevents subsequent progression of acute lung injury induced by endotoxin inhalation in hamsters. Am J Respir Crit Care Med 2000;161:2013–18 10852782 10.1164/ajrccm.161.6.9904047

[27] Looney MR Su X Van Ziffle JA Neutrophils and the Fc gamma receptors are essential in a mouse model of transfusion-related acute lung injury. J Clin Invest 2006;116:1615–23 16710475 10.1172/JCI27238 PMC1462945

[28] Matute-Bello G Martin TR Apoptosis in acute lung injury. Critical Care 2003;7:355–8 12974968 10.1186/cc1861 PMC270707

[29] Chollet-Martin S Montravers P Gibert C Subpopulation of hyperresponsive polymorphonuclear neutrophils in patients with adult respiratory distress syndrome. Role of cytokine production. Am Rev Respir Dis 1992;146:990–6 1416430 10.1164/ajrccm/146.4.990

[30] Zimmerman GA Renzetti AD Hill HR Functional and metabolic activity of granulocytes from patients with adult respiratory distress syndrome. Evidence for activated neutrophils in the pulmonary circulation. Am Rev Respir Dis 1983;127:290–300 6830052 10.1164/arrd.1983.127.3.290

[31] Worthen GS Schwab B Elson EL Mechanics of stimulated neutrophils: cell stiffening induces retention in capillaries. Science 1989;245:183–6 2749255 10.1126/science.2749255

[32] Haslett C Worthen GS Giclas PC The pulmonary vascular sequestration of neutrophils in endotoxemia is initiated by an effect of endotoxin on the neutrophil in the rabbit. Am Rev Respir Dis 1987;136:9–18 3605849 10.1164/ajrccm/136.1.9

[33] Tam FW Dixon CW Stuttle AW Inhaled platelet-activating factor causes pulmonary neutrophil sequestration in normal humans. Am Rev Respir Dis 1992;146:1003–8 1416388 10.1164/ajrccm/146.4.1003

[34] Doerschuk CM Beyers N Coxson H Comparison of neutrophil and capillary diameters and their relation to neutrophil sequestration in the lung. J Appl Physiol 1993;74:3040–5 8366005 10.1152/jappl.1993.74.6.3040

[35] Thakur ML Lavender JP Arnot RN Indium-111-labeled autologous leukocytes in man. J Nucl Med 1977;18:1014–21 409745

