# Supplementary material for: Pulmonary retention of primed neutrophils: a novel protective host response, which is impaired in the acute respiratory distress syndrome
Source: Thorax. 2014 Apr 4;69(7):623–9. doi: 10.1136/thoraxjnl-2013-204742 (PMC4055272; doi:10.1136/thoraxjnl-2013-204742)

SUPPLEMENTARY DATA

Supplemenary table 1

Absolute values for forward scatter, and neutrophil cell surface expression of CD11b and CD62L.

	Pre-operative controls	Sepsis	ARDS
Venous forward scatter	247.2 (219.4-282.9)	230.5 (214.1-265.1)	254.1 (194.1-288.4)
Arterial forward scatter	215.4 (208.8-262.2)	223.3 (210.5-260.0)	234.4 (198.6-369.1)
Venous CD62L (MFI)	119.2 (101.1-171.3)	30.75 (19.48-100.2)	28.85 (18.4-59.0)
Arterial CD62L (MFI)	150.6 (104-8-190.8)	37.95 (21.4-106.7)	27.05 (16.9-48.2)
Venous CD11b (MFI	40.73 (21.3-69.9)	62.72 (37.2-107.9)	64.2 (31.5-96.0)
Arterial CD11b (MFI)	31.83 (18.8-65.3)	70.19 (40.3-102.3)	70.5 (32.6-92.3)

Data shown as median (interquartile range).

MFI – mean fluorescence intensity

Figure legend for Supplementary figure 1

Paired samples of whole blood were obtained from the radial artery and internal jugular veins of a subject with sepsis secondary to Group A streptococcal bacteraemia who developed ARDS from day 2 onwards. Neutrophil cell surface expression of CD62L was measured using no lysis whole blood flow cytometry. Gradients were expressed as the ratio of the arterial value over the venous value. Supplementary figure 1 shows the changes in the CD62L gradient over time. P/F ratio is the partial pressure of oxygen in arterial blood / fraction of inspired oxygen.

Summers et al
Supplementary figure 1

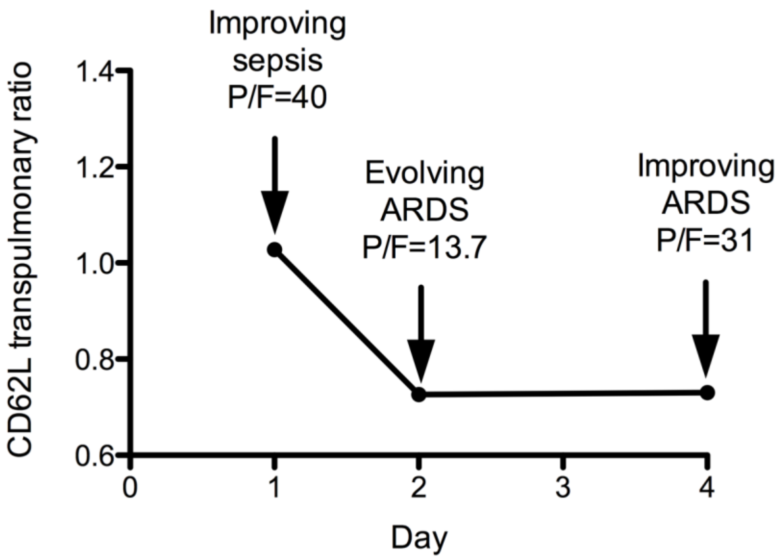

Supplement: Web supplement [file thoraxjnl-2013-204742-s1.pdf]
